# Pilates Reducing Falls Risk Factors in Healthy Older Adults: A Systematic Review and Meta-Analysis

**DOI:** 10.3389/fmed.2021.708883

**Published:** 2021-09-01

**Authors:** Larissa Donatoni da Silva, Agnes Shiel, Caroline McIntosh

**Affiliations:** ^1^School of Health Sciences, National University of Ireland Galway, Galway, Ireland; ^2^Department of Occupational Therapy, School of Health Sciences, National University of Ireland Galway, Galway, Ireland; ^3^Department of Podiatry, School of Health Sciences, National University of Ireland Galway, Galway, Ireland

**Keywords:** falls prevention, Pilates, balance, gait, functional mobility

## Abstract

**Background:** The main theme of this systematic review and meta-analysis is to synthesize the evidence of randomized controlled trial of evidence of Pilates intervention, in comparison to control groups and other forms of exercise, for falls prevention in healthy older adults.

**Methods:** The following electronic databases were searched up to October 2020; EMBASE, Scopus, Google Scholar, MEDLINE (Ovid), Science Direct, Cochrane, and CINAHL. The recommendations of the Preferred Reporting Items of Systematic Reviews and Meta-Analyses were followed. A PICOS approach was adopted as a framework to formulate the research question and set the inclusion and exclusion criteria. Participants were healthy older adults, defined as older adults who have maintained functional ability, including participants of both genders, those with a falls history, non-fallers, and individuals who were considered to be sedentary or active. Randomized controlled trials studies, written in the English language, from the decade, were included if they focused on specific outcome measures to decrease falls risk; functional mobility, mobility, fear of falling, gait, and postural stability. The PEDro scale was used to assess risk of bias.

**Results:** There were included 12 studies. In total, 702 healthy older adults' participants were included. Pilates showed an effect in mediolateral directions in comparison to control groups (MD = −1.77, 95% CI, −2.84 to −0.70, *p* = 0.001, heterogeneity: *I*^2^ = 3%), mobility (MD = 9.23, 95% CI, 5.74 to 12.73, *p* < 0.00001, heterogeneity: *I*^2^ = 75%) and fear of falling (MD = −8.61, 95% CI, −10.16 to −7.07, *p* < 0.00001, heterogeneity: *I*^2^ = 88%). In relation to other exercises group, Pilates showed positive effects in functional mobility (MD = −1.21, 95% CI, −2.30 to −0.11, *p* = 0.03, heterogeneity: *I*^2^ = 80%), mobility (MD = 3.25, 95% CI, 1.46 to 5.04, *p* < 0.0004, heterogeneity: *I*^2^ = 0%). No evidence of an improvement was found between the groups for dynamic gait index (MD = 2.26, 95% CI, −0.05 to 4.56, *p* = 0.06, heterogeneity: *I*^2^ = 86%), anteroposterior directions of balance (MD = −1.58, 95% CI, −3.74 to −0.59, *p* = 0.15, heterogeneity: *I*^2^ = 51%) and functional mobility when compared to control groups (no exercise) (MD = −1.24, 95% CI, −2.48 to −0.00, *p* = 0.05, heterogeneity: *I*^2^ = 87%).

**Discussion:** Pilates may be effective in decreasing the risk of falls in older adults. Pilates intervention was found to improve functional mobility, mobility, gait, fear of falling and postural stability and therefore there is some evidence to suggest that Pilates reduces certain risk factors for falls in healthy older adults. However, there is an absence of high-quality evidence in regards to the impact of Pilates on reducing falls and further robust RCTs are needed.

**Systematic Review Registration:** [PROSPERO], identifier [CRD42021206134].

## Introduction

A fall is “an unexpected event in which the participant comes to rest on the ground, floor, or lower level” [(1), p. 1618–22]. Falls are a leading cause of morbidity, mortality, functional deterioration, hospitalization, institutionalization, and pose a significant financial burden to health and social care services across the globe (2). It is estimated that, of the 646,000 deaths following falls each year, more that 80% occur in low and middle-income countries (3). The occurrence of falls is known to increase with advancing age; from 18% in young adults to 21% in middle age and 35% in older adults (4). The middle-aged population has been shown to have the highest percentage of injuries (70.5%) particularly in the knees, while older adults most frequently incur injuries from the head to the knee (4). In relation to gender, evidence suggests that women (20.1%) are more likely to fall than men (18.2%) (5). Thus, women have the highest frequency of injuries across all age groups (4).

The aging process is associated with decreased walking ability and walking speed. In individuals between the ages of 25 and 75 years, it is known that muscle power declines by 49% and muscle strength declines by 33% in this period (6). In older adults, the number of steps taken daily and walking speed were reduced by 75% between the ages of 60 and 85 and the falls per number of steps taken per day increases by 800% (6).

The Pilates method was developed in the 1920's by Joseph Hubertus Pilates and based on “Contrology” which aimed to coordinate the balance of the body, mind and spirit. The Pilates method also focused on concentration, strength and mobility (7). Pilates has been shown to improve lower limb muscle strength, static and dynamic postural balance and functional mobility after completion of a 12-week programme (8). Pilates intervention has also been shown to decrease the fear of falling in post-menopause women (9, 10), older adults (11), and in participants with low back pain (12). Other techniques such as Yoga and Tai Chi exercises have also been suggested to improve balance and prevent falls (13).

Several previous systematic reviews (14–18) have reported on the effectiveness of Pilates. The practice improves health status, balance, muscle strength, flexibility, functional autonomy, muscle endurance, body composition and aerobic endurance (16), functional capacity to perform daily living activities (14), and quality of life (8, 14, 18). A previous meta-analysis of Pilates included 10 studies with different subjects, such as healthy participants, those with a stable but chronic disease and Parkinson's disease. The analysis showed improvements in muscle strength and static and dynamic balance in older adults (14). Previous systematic reviews have investigated the improvement in balance after Pilates (14, 15, 18) and the prevention of falls in older adults (18, 19). However, specific task training has been shown to improve balance more than Pilates-only groups (18).

A recent meta-analysis of Pilates found improvement in postural stability in older adults (15, 20). The authors included randomized controlled trials (RCTs), quasi-experimental and crossover designs studies and found that only four out of 15 studies measured static balance. The author suggested that mat-based Pilates exercises should be performed for 40 min, three times per week for 5 weeks, or two or three times per week, to improve balance (15). However, Engers et al. (17) argued that Pilates studies must also be of good quality, feature control groups and follow-up and make use of the more rigorous randomized controlled trial methodology. Bueno et al. (21) suggest that more evidence is needed to judge the effects of mat Pilates on other physical functional measures in older adults.

The systematic reviews and meta-analyses are important because they summarize the empirical evidence and analyse the results of Pilates intervention studies. They summarize information with regard to the effectiveness of Pilates RCTs for health care professionals which, might help to inform them and their clinical practice of the benefits of Pilates interventions for older adults. However, previous meta-analyses on the effectiveness of Pilates in falls prevention have shown that studies are still lacking and there is no definitive evidence on Pilates interventions in reducing/ preventing falls. Furthermore, it is still unclear whether postural balance and gait can be improved with Pilates intervention. Regarding gait, there is a distinct lack of data concerning the potential impact of Pilates intervention on the spatiotemporal parameters of gait in healthy participants, and there is a dearth of evidence from RCTs and systematic reviews. Relating to balance, a previous meta-analysis did not separate the measures of postural balance for fall risk, such as mediolateral and anteroposterior parameters and fear of falling, in healthy participants. It is important to address and clarify these fall factors to reduce any knowledge gaps for future researchers.

Further improvements in the clinical practice of Pilates for specific age groups and guidelines are needed in the context of Pilates, since broader falls prevention guidelines are available. Therefore, it is necessary to include the following in meta-analyses: randomized clinical trials (RCTs) in evidence-based Pilates practice; falls protocol for longer follow-up; and recording falls during the intervention (to measure any reduction in the incidence of falls during the intervention group program). The research question asked whether Pilates training reduces the risk of falls in healthy older adults, defined as older adults who have maintained functional ability, including participants of both genders, those with and without a fall history and those considered sedentary or active.

The main theme of this systematic review and meta-analysis is to synthesize the evidence of RCTs of Pilates intervention in comparison to control group (no exercise) and to other exercises focuses on reducing the risk of falls by improving falls risk factors for the following outcome measures; mobility, functional mobility, fear of falling, gait, postural stability and falls recorded during the Pilates intervention.

## Methods

This systematic review and meta-analysis followed the general guidelines of the Preferred Reporting Items for Systematic Reviews and Meta-Analysis (PRISMA). The protocol for this systematic review registered in the International Prospective Register of Systematic Reviews (PROSPERO) number CRD42021206134.

### Eligibility Criteria

The studies selected met the following inclusion criteria using PICOS:

- Population (P): Healthy older adults 60 years of age and older (male and female).
- Intervention (I): All Pilates interventions, including mats, accessories and equipment.
- Comparators (C): A comparison of Pilates training with parallel groups, including a control group with no intervention and a control group with other exercises.
- Outcomes (O): Pre- and post-tests with regard to fear of falling, mobility, functional mobility, gait and postural stability by platform.
- Study design (S): RCTs and peer-reviewed publications written in the English language and dated between 2010 and 2020.

Not all studies included were necessarily aimed at evaluating the effects of Pilates in preventing falls in older adults. This was due to a lack of studies that investigate the effect of Pilates on falls prevention specifically.

The exclusion criteria were: participants with neurological impairment or orthopedic conditions such as lower back pain; the use of dynamic balance to evaluate balance and with no platform used for postural stability and non-RCT studies, such as semi- or quasi-experimental studies.

### Search Strategy

Electronic databases EMBASE, Scopus, Google Scholar, MEDLINE (Ovid), Science Direct, Cochrane and the Cumulative Index to Nursing and Allied Health Literature (CINAHL), were searched until 30th of October 2020.

The following search terms were used: Pilates AND healthy older adults, OR elderly OR aged, fall prevention OR risk of fall, fear of falling, postural balance OR balance, functional mobility, gait OR spatiotemporal parameters of gait AND randomized controlled trial.

### Study Selection

The Covidence systematic review component of Cochrane 1.0 extraction was used for importing citations, managing screening and data extraction by the reviewers (www.covidence.org).

The citations were imported into the Covidence systematic review software where any duplicate papers were excluded. Titles and abstracts were screened by two independent reviewers (LD and CM). Any disagreements between the reviewers were mutually resolved to reach a consensus. Potentially eligible articles were then reviewed in full text by two authors (LD and CM) and any disagreements were mutually resolved to reach a consensus.

### Data Extraction

Data were extracted independently by two reviewers (LD and CM) in Covidence. Consensus was reached at a later meeting between the two authors. Data extracted included participant demographics (age and sample size), study details (author, year, country), study design, setting or recruitment, aim, intervention groups and inclusion criteria. Pilates intervention description (material, duration and times per week of intervention), participants analyzed, findings and recommendations.

### Outcome Measures

The primary and secondary outcomes selected are associated with a decreased fall risk in older adults. Functional mobility: The TUG test is a sensitive and specific tool to identify community-dwelling adults who are at risk of falling, including older adults who have balance impairments and who live independently within a community. Older adults who scored ≥ 13.5 s to perform the TUG were classified as fallers with an overall accurate prediction rate of 90% (22).

The parameters of postural stability: The anteroposterior parameter was associated with a history of falls for the conditions of eyes opened and eyes closed on a firm surface (23). Impaired balance in the lateral direction was related to a risk of falls (24), while the mediolateral displacement of the center of pressure was associated with future falls (25).

Tasks related to gait changes have been identified as fall predictors (26) among older adults with FOF without normal gait (27). Gait speed is a simple and fast variable for measuring fall risk (28) and functional capacity for health outcomes in community-dwelling older adults (29).

Primary outcomes included functional mobility (the Timed-Up-and-Go-TUG task), mobility as the functional reach test (FRT), fear of falling (the 16-item Falls Efficacy Scale-FES, questionnaire) and postural stability by force platform using COP displacement to evaluate the parameters in mediolateral (ML) and anteroposterior (AP) directions under both eyes open and closed conditions.

Secondary outcomes included falls in the past year (within 12 months), the number of falls recorded during the study, gait (the 10-min walk test- 10 MWT and the 6-min walk test- 6 MWT) and the Dynamic Gait Index (DGI). In case of incomplete or missing data for spatiotemporal parameters of gait for RCTs in healthy subjects.

### Quality Assessment

The risk of bias in assessing the quality of the included studies was evaluated by two independent reviewers (LD and CM). Consensus was reached at a later meeting between the two authors.

The inclusion criteria were evaluated using the Database of Physiotherapy Evidence (PEDro) scale (http://www.pedro.org.au/english/downloads/pedro-scale) for RCTs, which contained 10 questions to assess the study quality. A study score of 6–10 is considered moderate to high quality and a score <5 is considered lower quality according to their guidelines (http://www.pedro.org.au/english/downloads/pedro-statistics/). Consensus was reached by the two independent graders and there was no requirement for a third reviewer to resolve disagreements.

### Data Analysis

Statistical analyses were performed using the software package Statistic 10.0 and Cochrane Review Manager Software (RevMan 5.4, Cochrane Collaboration). A value of α = 0.05 was considered statistically significant.

Data were entered in the software as mean and standard deviation (SD) and the total number of participants in each study allocated into groups. The author considered whether the studies reported on whether or not intention- to- treat analysis was used.

Data reported as standard errors or confidence intervals (CIs) were converted to SD using https://training.cochrane.org/handbook/current/chapter-06#section-6-5-2. If the extracted data were incomplete, the author was contacted by email for more details.

Continuous data outcomes were reported as the mean difference (MD) were reported with 95% CIs. Postural stability included two subgroups to evaluate the estimating effects for the variables for mediolateral directions (MLEO; MLEC) and for anteroposterior directions (APEO; APEC). Assuming pooled effects, a fixed-effects model with heterogeneity *I*^2^ ≤ 50% and a random-effects model with *I*^2^ ≥ 50% were used. Forest plots presented the comparison between the Pilates intervention group and the control group with no exercise. The variables TUG and FRT were also analyzed for Pilates vs. other exercise groups. Dichotomous data for the number of falls in participants during the previous year and the number of falls during the intervention were reported as exploratory due to the lower reporting of data during the intervention programme.

## Results

### Study Selection

**Figure 1:**
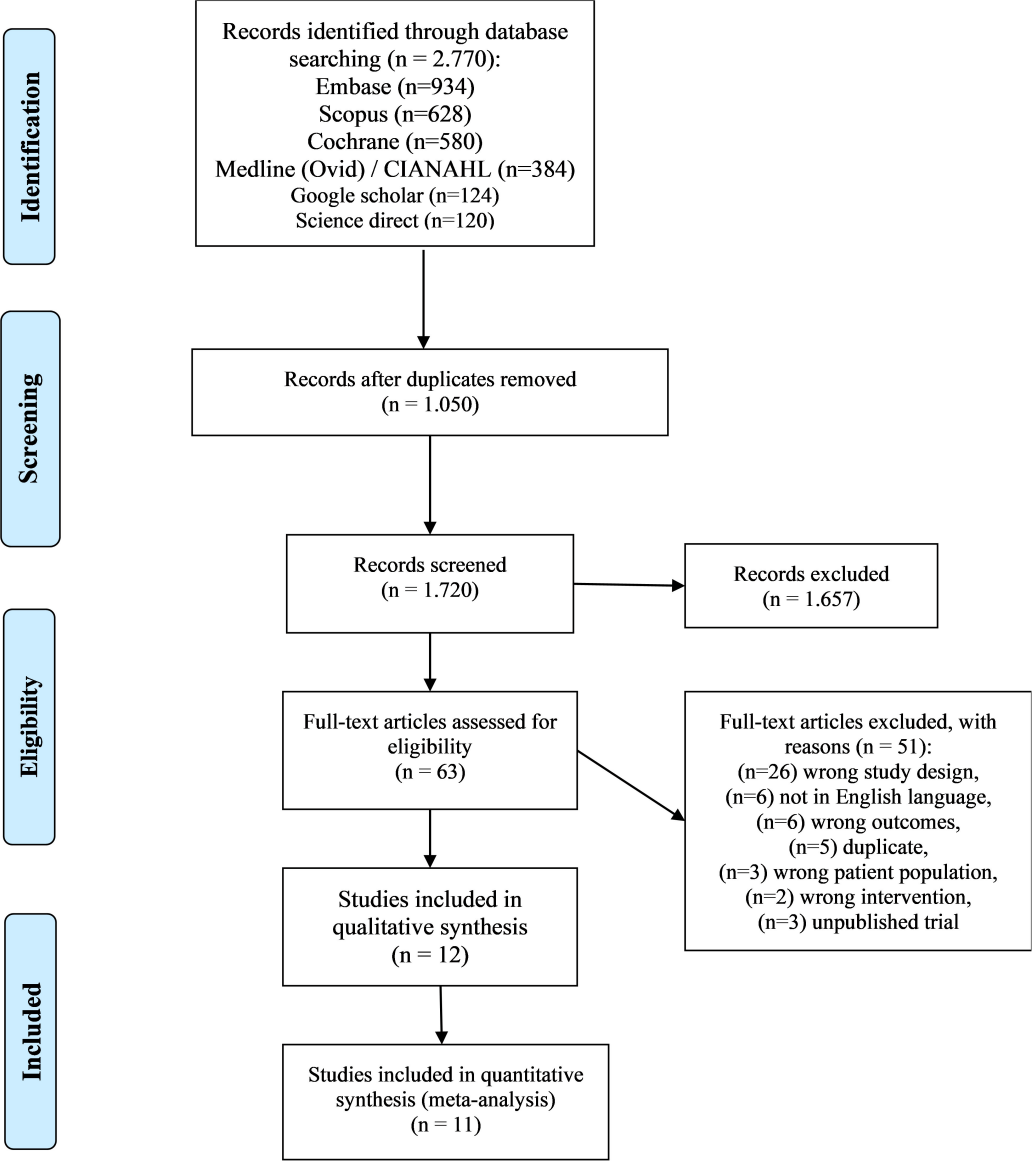
Studies included.

A total of 1,720 records were screened and 1,657 were excluded. A total of 63 studies were assessed for full-text eligibility and 51 were excluded. Twelve RCT studies were identified after the selection process for systematic review and meta-analysis; one study was excluded as the authors included path length variable as opposed to ML and AP variables of balance (30) (see Figure 1). The studies were conducted between 2012 and 2019.

### Participants

In total, 702 participants were included, with 308 allocated to Pilates group (PG), 316 to Control group, (CG) and 78 to the three-arm exercise group.

Nine studies included both men and women (11, 30–37), while three (8, 10, 38) included only women.

Participants were healthy older adults, defined as older adults who have maintained functional ability (WHO). Six studies included healthy participants (10, 11, 30, 31, 34, 36). Other studies have inclusion criteria with restrictions such as sedentary women (8, 38) and fallers were included (32, 33, 35, 37).

At baseline, six studies (10, 11, 33–35, 37) reported participants who fell in the past year (*n* = 338 participants), shared between the Pilates group (*n* = 168) and control group (*n* = 170).

Five studies (10, 11, 33–35) reported the number of fallers (*n* = 283 participants) in the previous year at baseline before being allocated to the intervention programme. In the Pilates group (*n* = 141 participants), the number of fallers was 49 and in the control group (*n* = 142 participants), the number of fallers was 43. One study reported no events (falls) in the control group (34). Only two studies (33, 35) reported the number of falls during the intervention programme.

Two studies (35, 37) used TUG test scores ≥ 13.5 s to screen participants with a history of falls. Josephs et al. (35) also used Advanced Balance Scale (FAB) > 25 and Activities-Specific Balance Confidence Scale (ABC). Surbala et al. (32) used a FES-16 item score > 23 to screen participants.

The criteria for participants included a risk of sustaining a fall injury (33), having two or more falls or one injurious fall in the previous year (37), having at least one fall in the previous year (32), having a fall in the past year or cut-off points for TUG or FAB, or able to complete the questionnaires without assistance (35).

### Study Characteristics

Of the 12 studies, six compared Pilates intervention to a control group with no exercise (10, 11, 31, 33, 36, 37). One crossover study (31) was included (see Table 1).

**Table 1:** Study and participants characteristics.

**References/Country**	**Design**	**Setting or recruitment**	**Aims**	**Inclusion criteria**	**Total sample size (n), and between intervention groups**	**Age: Mean (SD) between groups**
Aibar-Almazán et al. (10) Spain	RCT	Community-dwelling postmenopausal women	To analyse the effects that an exercise programme based on the Pilates method would have on women aged 60 years and older concerning their postural control, FOF, and balance confidence when performing daily activities.	Women, aged 60 years and over and with at least 12 months since their final menstrual period, not involved in a Pilates exercise programme in the last year; and physically independent enough to perform basic daily activities (39)	*Total, (n = 110)* PILATES, PG *(n = 55)* / NO INTERVENTION, CG *(n = 55)*	PG = 69.98 (7.83) CG = 66.79 (10.14)
Barker et al. (33) Australia	RCT	Advertisements in local general practitioner (GP), imaging and physiotherapy clinics; university newsletters; local community centers and newspapers.	To conduct a pilot, single blinded RCT to assess the feasibility of a Pilates exercise program that incorporates best practice guideline recommendations for falls prevention exercise and obtain a preliminary estimate of effect of the program on falls, fall injuries and fall risk factor outcomes to inform the design of a larger clinical trial.	aged ≥ 60 years; at risk of sustaining a fall injury based on a telephone screen developed by the research team (Box 1, available online); and able to negotiate a set of 10 stairs independently without a gait aid.	*Total, (n = 53)* PILATES PG *(n = 22)* / NO INTERVENTIONCG, *(n = 31)*	PG = 69.25 (6.74) CG = 69.41 (5z.76)
Bird et al. (31) Australia	RCT (crossover)	Local community groups in an urban area using radio and print media	To conduct a randomized controlled trial to investigate the effects of a Pilates intervention on the variables of static and dynamic balance and leg strength in a group of community-dwelling adults older than 60 years.	Participants did not currently have or had not recently had an acute medical condition. Volunteers who had controlled chronic conditions such as arthritis or stable chronic cardiovascular or metabolic conditions (e.g., hypertension, diabetes mellitus) were included in the study.	*Total, (n = 32)* PILATES, PG *(n = 17)* / NO INTERVENTION, CG *(n = 15)*, at crossover PG *(n = 14)* CG *(n = 13)* (daily activity monitored by (CHAMPS) questionnaire)	No mentioned
Oliveira et al. (8) Brazil	RCT	Community-dwelling older adults in the city of Jacarezinho State of Paraná, Brazil	To determine the isokinetic torque of the knee extensors and flexors, static and dynamic balance, functional mobility, and quality of life of community-dwelling older adults who performed a Pilates exercise protocol.	Age 60–65 years; female; the ability to perform basic and instrumental activities of daily living without assistance; a body mass index (BMI) within the ideal range for the age group (22–27 kg/m^2^); a statement from a physician indicating sufficient fitness for the practice of physical exercises; not having practiced any type of physical exercise in the previous 6 months; and agreement not to participate in any other type of physical exercise during the study	*Total, (n = 32)* PILATES, PG *(n = 16)* / STATIC STRETCHING, CG *(n = 16)*	PG = 63.6 (1.0) CG = 64.2 (0.8)
Donath et al. (30) Switzerland	RCT 3 arms	Community-dwelling seniors	Investigating whether the neuromuscular training effects are in favor of a traditional balance training program (BAL) or a mat-based Pilates training (PIL) in a group of healthy community-dwelling seniors	healthy seniors (75% women) without artificial joints, neurological and internal diseases, osteoporosis, acute and chronic back pain as well as trauma and balance or strength training experience within the last 6 months	*Total, (n = 59)* PILATES, PG *(n = 20)* / MULTIMODAL BALANCE TRAINING, BAL *(n = 20)* / NO INTERVENTION, CG *(n = 19)*. (daily activities)	PG =70.8 (6.5) BAL = 69.1 (5.8) CG = 69.2 (6.1)
Gabizon et al. (34) Israel	RCT	Mail Community-dwelling, independent older adults were recruited from Lehavim, a community with a high social-economic ranking near Beer-Sheva, in southern Israel	To assess whether a Pilates-training program that includes classical Pilates exercises and exercises using Thera-Band elastic resistance bands and Swiss balls would improve balance control parameters associated with an increased risk of falling.	65 years of age or older, could ambulate independently (i.e., use of a cane was acceptable, but not a walker), did not have severe focal muscle weakness or visual impairment, did not have known neurological disorders (including stroke or Parkinson's disease), did not have metastatic cancer, and did not take medications that impair balance or strength. All subjects provided a medical waiver, signed by their primary care physician, clearing them to participate in moderate physical exercise.	*Total, (n = 88)* PILATES with balance, PG *(n = 44)* / NO INTERVENTION, CG *(n = 44)*	PG =70.3 (3.8) CG =72.1 (4.60)
Josephs et al. (35) USA	RCT	Local physicians in the area, seniors' groups at churches and community centers, word of mouth and notices posted in the local libraries	To investigate the effectiveness of Pilates group exercise vs. traditional strength and balance group exercise for improving balance, reducing falls and improving balance confidence in community dwelling older adults with fall risk	65 years of age or older living in the community; impaired balance as defined by at least one of the following: a fall in the past year, TUG > 13.5 s or FAB ≤ 25; and ability to follow instructions as assessed by the ability to complete the questionnaires without assistance. Subjects were not screened for ability, such as use of assistive device for walking, but only that they met the inclusion criteria of history of fall or meeting the cut off for balance compromise with the TUG or FAB.	*Total, (n = 31)* PILATES / TRADITIONAL STRENGHT AND BALANCE No mentioned	PG = 75.6 (6.2) TG = 74.5(6.9)
Mesquita et al. (38) Brazil	RCT 3 arms	Older women belonging to a church project	To conduct a randomized controlled trial to investigate and compare the effect of both exercise methods on the static and dynamic postural balance variables in elderly women, thus identifying alternatives to prevent falls and promoting functional independency	Women who were sedentary as evaluated using the International Physical Activity Questionnaire and aged 60–80 years were included in the sample	*Total, (n = 63)* PILATES, PG *(n = 21) /* PNFG (*n* = 21) / NO INTERVENTION, CG (*n* = 21) (daily activity)	PG = 67.3 (4.9) PNFG = 68.5 (5.4) CG = 71.5 (6.2)
Roller et al. (37) California	RCT	Core Conditioning in Studio City advertisements in newspapers and at senior centers, and by word of mouth, and were screened *via* a telephone	To investigate whether Pilates Reformer exercises would improve balance, reduce fall risk, improve functional mobility, and improve balance confidence in adults age 65 and older at risk for falls.	Aged 65 years or older, self-reported history of two or more falls or one injurious fall in the past year, TUG test score of 13.5 s suggesting risk for falling and physician approval to participate in the study.	*Total, (n = 59)* PILATES, PG *(n = 27)* / NO INTERVENTION, CG *(n = 28)*	PG = 78.52 (7.57) CG = 76.68 (6.79)
Surbala et al. (32) India	RCT 3 arms	Ambulatory geriatric subjects were recruited from four different old age homes (OAH) in Surendranagar area	The study aims in determining and comparing the effectiveness of PI and CBT specially designed for the elderly population in improving functional balance and QOL.	Age between 65 and 74 years both males and females; able to walk at least 30 feet with or without an assistive device; not participating in any sports or physical therapy sessions; willingness to do physical exercise thrice a week with regular attendance; have fallen at least once within previous year; fear of fall scoring >23 in 16 items falls efficacy scale international questionnaire; Mini-Mental Status Examination score of 24; and no affirmative responses to the PAR-Q instrument for inactive older adults	*Total, (n = 51)* PILATES PG (*n* = 17) / CONVENTIONAL BALANCE TRAINING, CBT *(n = 17)* / CONTROL, CG *(n = 17)*. (exercise)	PG = 70.7 (2.7) CBT =70.3 (2.9) CG = 69.35 (3.0)
Vieira et al. (36) Brazil	RCT	Community dwelling seniors	To investigate the effects of a 12-week Pilates-inspired exercise program on functional performance among community-dwelling older women	Each subject had been instructed to avoid caffeinated and alcoholic beverages and to not perform moderate or heavy exercise the day before and the day of the application of the protocols. Before beginning the test, subjects were interviewed and examined to confirm their good health and whether they had a normal night's sleep	*Total, (n = 52)* PILATES, PG *(n = 26)* / NO INTERVENTION, CG *(n = 26)* (daily activity)	PG = 66.0 (1.35) CG = 63.3 (0.91)
Badiei et al. (11) Iran	RCT	Elderly women who were referred to the day care center of Kahrizak sanatorium (Alborz Province) *via* the convenient sampling method.	To determine the effect of Pilates exercise on Fear of Falling (FOF) among elderly women	Age between 60 and 80 years, willingness to join the study and signing the consent form, having medical approval that certifies the person's ability to participate in physical activity and exercise routines, no history of hospitalization in the past 3 months as well as ability and availability to attend at least 80% of the Pilates exercise sessions.	*Total, (n = 44)* PILATES, PG *(n = 22)* /NO INTERVENTION, CG *(n = 22)* (daily activity - (stretching training) as usual.	PG = 68 (5.9) CG =71 (4.1)

*RCT, Randomized Controlled Trial; PG, Pilates group; CG, control group; CBT, Conventional Balance Training; PNFG, Proprioceptive neuromuscular facilitation group; PI, principal investigator; OAH, old age homes; TUG, Time Up and Go; FAB, Frontal assessment battery; TG, Traditional strength; BAL, traditional balance training program; PIL, Pilates training; BMI, Body mass index; GP, general practitioner; FOF, Fear of falling; QOL, Quality of life; PAR-Q, Physical Activity Readiness Questionnaire*.

Five studies compared Pilates intervention to other interventions, such as static stretching (8) and traditional strength and balance (35). Three three-arm studies were included to compare Pilates intervention to the following: conventional balance training and control (exercise) (32); Pilates neuro-proprioceptive facilitation group (PNFG) and no intervention (daily activities) (38); and multimodal balance training and no intervention (daily activities) (30).

One study aimed to prevent falls (33), one looked at alternatives to prevent falls (38), and one aimed to reduce falls (35).

### Risk of Bias Within Studies

Of all the RCTs, only one included the highest quality score (10). Seven studies were of moderate to high quality of 6–10 scores (8, 30–34, 38); four studies scored lower (11, 35–37).

Five studies did not have concealed allocation (11, 30, 35, 37, 38). Three studies were not similar at the baseline (11, 34, 35). Only one study was blinded from subjects (10). Two studies were blinded from therapists (10, 30). Four studies were unblinded to the assessors (8, 11, 30, 36). Three studies did not have appropriate follow-up (31, 34, 36). Seven studies used intention to treat analyses (8, 10, 11, 31–33, 37). Only one study did not compare between group analysis (32) (see Table 2).

**Table 2:** Quality of assessments of include studies.

**References**	**Random allocation**	**Concealed allocation**	**Similar at baseline**	**Blinding of subjects**	**Blinding of therapists**	**Blinding of assessors**	**Follow-up**	**Intention to treat analysis**	**Comparison between groups**	**Point measures variability**	**Score**
Aibar-Almazán et al. (10)	Y	Y	Y	Y	Y	Y	Y	Y	Y	Y	10/10
Barker et al. (33)	Y	Y	Y	N	N	Y	Y	Y	Y	Y	8/10
Bird et al. (31)	Y	Y	Y	N	N	Y	N	Y	Y	Y	7/10
Oliveira et al. (8)	Y	Y	Y	N	N	N	Y	Y	Y	Y	7/10
Donath et al. (30)	Y	N	Y	N	Y	N	Y	N	Y	Y	6/10
Gabizon et al. (34)	Y	Y	N	N	N	Y	N	N	Y	Y	6/10
Josephs et al. (35)	Y	N	N	N	N	Y	Y	N	Y	Y	5/10
Mesquita et al. (38)	Y	N	Y	N	N	Y	Y	N	Y	Y	6/10
Roller et al. (37)	Y	N	Y	N	N	Y	Y	Y	Y	Y	7/10
Surbala et al. (32)	Y	Y	Y	N	N	Y	Y	Y	N	Y	7/10
Vieira et al. (36)	Y	Y	Y	N	N	N	N	N	Y	Y	5/10
Badiei et al. (11)	Y	N	N	N	N	N	Y	Y	Y	Y	5/10

### Interventions

This study focused on the PICO method. The intervention included all types of Pilates methods, but the variables “intensity,” “dose,” and “type of Pilates method” could not be considered in the meta-analysis The strength of this study design is in its synthesisation of the results from the RCTs and the outcomes of fall risk factors. (*It is not always feasible to conduct randomized trials of all intervention types [e.g., the “structural” interventions mentioned in Section 17.2.3]* [cited in Cochrane interventions handbook Section 17.2.5.).

#### Period of Pilates Exercise

The Pilates intervention period was from 4 to 24 weeks. The 12-week period was more common among the studies (8, 10, 34–36) (see Table 3).

**Table 3:** Pilates exercise intervention, outcomes measures, participants analyzed and recommendations.

**References**	**Pilates intervention**	**Outcome measures of interested**	**Participants (*n*) analyzed intention to treat (IT)**	**Recommendations**
Aibar-Almazán et al. (10) Spain	12 weeks / 2 sessions per week / 60 min The last sessions involved equipment such as resistance bands, rings, and balls.	International Falls Efficacy Scale- FES-16 Force platform Number of participants falls in the past year, *n (%)* Pilates, 25 (45.45%) Control, 17 (32.69%)	IT- yes PG = 55 CG = 52	Future studies should consider the mid- and long-term effects, on both men and women, of the intervention here described.
Barker et al. (33) Australia	24 weeks / 2 sessions per week / 60-min Pilates class practice guidelines for exercise to prevent falls (educational letter). Home exercises (20 min) >50 h over total study period Standing exercises. Equipment: reformer, trapeze, Wunda chair, chi ball, elastic band and foam roller.	Functional reach test- FRT Timed Up and Go- TUG Dynamic gait index -DGI Number of participants falls in the past year, *n (%)* Pilates, 6 (30%) Control, 9 (38%) Number of Falls Pilates, *n* = 13 Control, *n* = 11	IT- yes PG = 20 CG = 29, and CG = 24 (fall)	Pilates exercise is an enjoyable and acceptable form of exercise in community-dwelling older people at risk of falling. An appropriately designed Pilates exercise program appears to improve standing balance and reduce the risk of falls. Based on the fall injury rates estimated here, we can estimate (with 80% power) that a future definitive study would require 402 participants per arm to detect a 30% difference in fall injury rates. These estimations are based on a negative binomial distribution and a 6-month follow-up period. A large RCT that includes around 804 people is warranted to confirm effects.
Bird et al. (31) Australia	5 weeks / 2 sessions per week / 60 min After a 6-week washout period, participants perform the alternate intervention. Classes consisted of standing exercises / Pilates reformer and mat-based exercises. Home-based with a diary	Force platform The Timed Up and Go –TUG	IT- yes PG = 27 CG = 27	Although there were no between-condition differences between the Pilates and control conditions, significant improvements were observed in the pooled static and dynamic balance data from the 2 Pilates conditions. The reported improvements in mediolateral sway range and dynamic balance may have positive functional implications for physical fall risk factors in an older population.
Oliveira et al. (8) Brazil	12 weeks / 2 sessions per week /60 min Equipment: combo chair, Cadillac trapeze table, universal Reformer and ladder barrel. All exercises were performed with one set of 10 repetitions. The Borg CR10 scale 21 was used to determine the level of effort and load progression.	The Timed Up and Go-TUG -	IT – yes PG = 16 CG = 16	Further studies are needed to determine the effects of Pilates for older adults. Based on the present findings, Pilates performed with equipment elicits improvements in lower limb muscle strength, static and dynamic postural balance, functional mobility and quality of life of older adults when performed in two weekly sessions for 12 weeks.
Donath et al. (30) Switzerland	8 weeks/ 2 sessions per week/ 60 min Mat Pilates 6–12 repetitions were performed during each exercise.	Force Platform	No- IT PG = 17 BAL = 16 CG = 15	Pilates training did not cause relevant adaptations. Future studies may also observe specific adaptations in neuromuscular, cognitive function and psychosocial health parameters could be assessed upon Pilates training, e.g., in frailer and residential seniors. In these cases, randomized controlled three armed study designs are recommended. Accordingly, any control condition should then consider appropriate group allocation and social gatherings, in order to avoid socially confounding situations.
Gabizon et al. (34) Israel	12 weeks / 3 times a week / 60 min Classical Pilates method with Thera-Band elastic resistance bands and Swiss balls	Force Platform Number of participants falls in the past year, *n (%)* Intervention 3 (6.8) Control 0 (0.0)	No-IT PG = 44 CG = 44	Further research should be conducted to assess the potential effect of Pilates training on a population of weaker older adults who have a history of falls.
Josephs et al. (35) USA	12 weeks/ 2 times week / 60 min Pilates with Reformer, Cadillac and Chair apparatus. Each exercise 10 repetitions. The traditional group: elastic resistance bands, ankle weights, foam balance pads, boxes of varying heights and half foam rollers were props performed 20 repetitions. Home exercises 15–20 min. Monthly calendar to record their home exercise participation.	The Timed Up and Go-TUG Number of participants falls in the past year: Pilates, *n* = 10 Traditional, *n* = 8 Number of falls Mean (SD) Pilates= 1.5 (1.3) ranged 0–4 Traditional = 1.8 (2.2) ranged 0–7	No- IT PG = 13 CG = 11	Future research ideas include having three groups, Pilates, traditional and a control group and following the results longer term. This study indicates that balance and balance confidence can be improved in <50 h in patients with fall risk. A future research study should investigate this further in adults with fall risk.
Mesquita et al. (38) Brazil	4 weeks / 3 times a week/ 50 min Mat Pilates, with Swiss ball, TheraBand, and magic circle.	Force platform The Timed Up and Go-TUG Functional reach test -FRT	No-IT PG = 20 CG = 18 PNFG = 20	Recommend that further studies include larger samples of elderly women and greater numbers of sessions. This will help to elucidate the optimal alternatives that can be applied to increase balance, allowing PNF and Pilates exercises to be used not only for rehabilitation, but also as a preventive method.
Roller et al. (37) California	10 weeks / Once a week / 45-min Pilates with Reformer 10 repetitions each, using progressive resistance of 2–4 springs Falls diary	The Timed Up and Go- TUG 10 Min walk test-10 MWT Number of participants falls in the past year Mean (SD) Pilates = 2.00 (2.30) Control = 3.21 (5.57)	IT- yes PG = 27 CG = 28	Future studies examining the effect of Pilates Reformer exercises on balance, gait, and fall risk in older adults may also want to consider performing exercises that work specifically on balance in upright postures such as standing on a moving carriage
Surbala et al. (32) India	6 weeks / 2 times per week/ 45 min Mat Pilates, ball exercise and in standing position. All exercises were done for 10 repetitions with a rest period of 2 min before commencing the next exercise.	Functional reach test -FRT The Timed Up and Go-TUG Dynamic Gait Index-DGI	IT- yes PG = 17 CBT = 17 CG = 17	Future research with cross over designs may also be conducted to determine the participants' preference of exercise program between PI and CBT. Further controlled comparative studies with larger sample size are recommended in community dwelling old elderly (over 75 years) individuals and those with pathological conditions (e.g., Stroke, Parkinsonism, etc.) who are at higher risk of falls and falls related injuries
Vieira et al. (36) Brazil	12 weeks / 2 times per week / 60 min Mat Pilates using accessories such as exercise rubber, bands, swiss and exercise balls.	Timed Up and Go-TUG 6-min walk test-6 MWT	No – IT PG = 21 CG = 19	Pilates-inspired exercises improved dynamic balance, lower-extremity strength, and cardiovascular fitness in community-dwelling older women. Therefore, it might be a potentially effective exercise regimen to maintain physical fitness and, possibly, to prevent disability and falls in old age. Yet, further investigation is needed to evaluate the effectiveness of the Pilates method on functional and physical fitness of older adults with characteristics that differ from those of our sample.
Badiei et al. (11) Iran	8 weeks / 3 times per week / 60 min Mat Pilates by Pérez et al. (40)	Falls Efficacy Scale- FES-16 ITEM Number of participants falls in the past year Mean (SD) Pilates = 1.54 (1.79) Control = 2 (2.4), *p* = 0.4 Pilates, *n (%)* YES 15 (68.2) NO 5 (22.7) Control*, n (%)* YES 17 (77.3) NO 7 (31.8), *p* = 0.5	IT- yes PG = 22 CG = 22	The findings of the present study can help in creating a new attitude toward the possible roles of exercising in decreasing the risk of falling and other related factors in the elderly population, especially elderly women. In addition, health care providers can use this study to formulate similar interventional strategies that can improve the quality of life of the elderly.

*n, Number; %, percentage; IT, Intention to treat; SD, Standard Deviation; TUG, Timed Up and Go; 6 MWT, 6-minute walk test; FRT, Functional Reach Test; DGI, Dynamic Gait Index; 10 MWT, 10 Minute walk test; FES-I, International Falls Efficacy Scale; RCT, Randomized controlled trial; PG, Pilates group; CG, control group; CBT, Conventional Balance Training; PI, principal investigator; TUG, Time Up and Go; PNF, Proprioceptive neuromuscular facilitation*.

The following studies had different periods of interventions: Mesquita et al. (38) 4 weeks; Bird et al. (31) 5 weeks; Surbala et al. (32) 6 weeks; Donath et al. (30) and Badiei et al. (11) 8 weeks; and Roller et al. (37) 10 weeks. The study by Barker et al. (19) included three analyses at baseline, after 12 weeks and after 24 weeks; the 24-week period was the longest follow-up period that recorded falls.

#### Number of Sessions

One to three Pilates sessions were held per week for 45–60 min each.

Eight studies (8, 10, 30–33, 35, 36) included two sessions per week for 60 min each. Three studies (11, 34, 38) included three sessions per week for 50 min each. Only one study included a single session per week lasting only 45 min (37).

#### Materials

Two studies included only mat Pilates (11, 30); Badiei et al. (11) used the programme from Pérez et al. (40).

Six studies included mat Pilates with accessories (10, 32, 34, 36, 38); one was in the traditional group (35).

Three studies included the use of Pilates equipment (8, 35, 37), which Josephs had two Pilates groups (accessories vs. equipment).

Three studies used a mix of Pilates techniques, including the mat with accessories, standing position (31–33) and equipment (31, 33).

Three studies included supplementary at-home exercises (31, 33, 35). Barker et al. (33) included educational materials for falls prevention and suggested that participants should exercise for 20 min on a daily basis. At-home Pilates exercise with mat were performed occasionally each week (31) participants performed the exercise for 15–20 min on the day that there was no intervention program and after the program ended, participants continued to do the exercises for 8 weeks (35).

### Outcomes

The outcomes are focused on reducing fall risk for the following outcome measures: history of falls, mobility, functional mobility, fear of falling, gait, and postural stability.

A total of 565 participants were included in the meta-analysis, with 282 allocated to PG and 283 to CG. A third exercise group included 37 participants.

#### Number of Fallers

Only Josephs et al. (35) (*n* = 31 participants) compared Pilates vs. the traditional group (other exercise group), allocating *n* = 10 fallers to the Pilates group and *n* = 8 fallers to other exercise group.

Barker et al. (33) was the only study to report the CI; no statistical significance was found—fallers *n* (%) = 7.5% (95% CI, −20.40 to 35.40, *p* = 0.601).

Roller et al. (37) reported the number of fallers as mean (SD) = 2.00 (2.30) in the Pilates group and as mean (SD) = 3.21 (5.57) in the control group.

#### Number of Falls During the Intervention Programme

Josephs et al. (35) reported that the number of falls in the Pilates group was 0–4, with Mean (SD) = 1.5 (1.3). In the traditional group, the number of falls was 0–7, with mean (SD) = 1.8 (2.2) and *p* = 0.703; no statistical significance was found.

Barker et al. (33) reported the total number of falls in the Pilates group (*n* = 13) and control group (*n* = 11) during the 24-week follow-up intervention programme. This study was the only one to report the rate of falls per 1,000 person-days across groups, which was calculated as the difference incidence rate ratio = 1.17 (95% CI, 0.43–3.16, *p* = 0.0754). They also stated that *n* = 2 falls (10%) occurred during the Pilates classes.

#### FES

**Figure 2:**
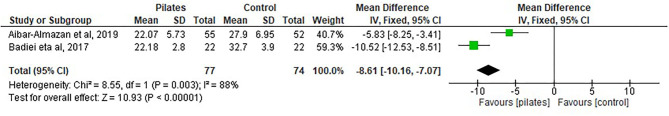
Fear of falling.

Two studies (10, 11) included the Pilates group (*n* = 77) and the control group with no intervention (*n* = 74). Badiei et al. (11) and Aibar-Almazán et al. (10) used the FES of a 16-item questionnaire on the fear of falling (see Figure 2).

The results show a decreased fear of falling score and statistically significant between groups in favor of the Pilates group: (MD = −8.61, 95% CI, −10.16 to −7.07, *p* < 0.00001, heterogeneity: *I*^2^ = 88%).

#### Postural Stability

Four studies (10, 31, 34, 38) included two subgroups for mediolateral directions (MLEO; MLEC) (*n* = 516) and two subgroups for anteroposterior directions (APEO; APEC) (*n* = 408) in the meta-analysis. All participants (*n* = 924) were allocated to the Pilates (*n* = 450) and control (no intervention, *n* = 474) subgroups. The three-arm study (Pilates vs. other interventions) by Mesquita et al. (38) was excluded. Other studies have not compared the Pilates to other interventions.

Participants performed the balance test on a firm surface (platform) for 30 s in all the included studies. The studies included balance bipedal performance (38), quiet standing trials with eyes open and more than 10 s with eyes closed /blindfolded (34) and the Romberg test (10).

#### Mediolateral

**Figure 3:**
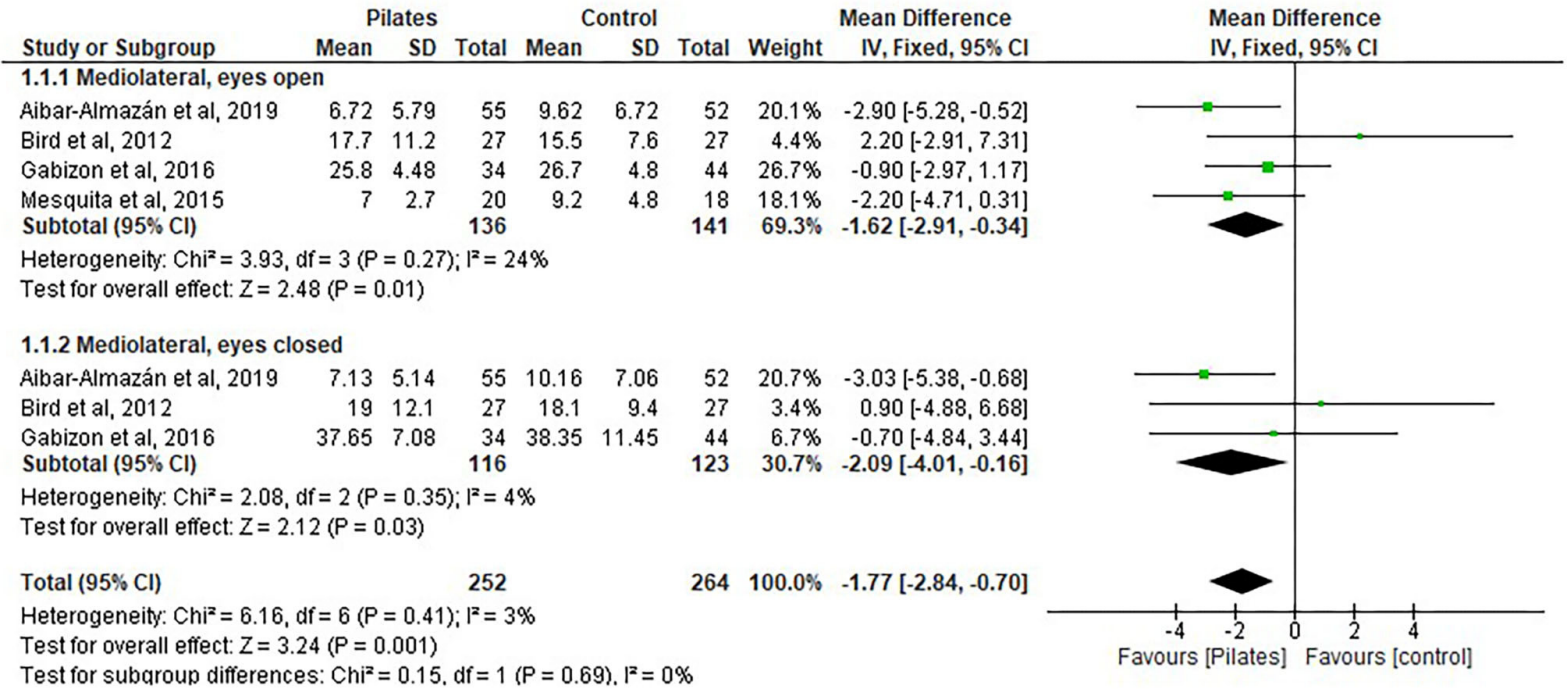
Postural stability-mediolateral.

Of the 516 participants included in this analysis, 264 were assigned to the Pilates group and 264 to the control group. The pooled overall balance improved, as seen in a decrease in scores after Pilates and was statistically significant between groups in favor of the Pilates group: (MD = −1.77, 95% CI, −2.84 to −0.70, *p* = 0.001, heterogeneity: *I*^2^ = 3%)—the postural stability of the subjects increased (see Figure 3).

The following four subgroups were shown separately on the forest plots:

MLEO: Four studies (10, 31, 34, 38) included the Pilates group (*n* = 136) and the control group with no intervention (*n* = 141). MLEO showed improvement in controlling postural stability with a decrease in the score and significant differences were found between the groups in favor of Pilates intervention: (MD = −1.62, 95% CI, −2.91 to −0.34, *p* = 0.01; heterogeneity: *I*^2^ = 24%).
MLEC: Three studies (10, 31, 34) included the Pilates group (*n* = 116) vs. the control group with no intervention (*n* = 123). MLEC showed improvement in controlling postural stability with a decrease in the score and significant differences were found between the groups in favor of Pilates intervention:(MD = −2.09, 95% CI, −4.01 to −0.16, *p* = 0.03, heterogeneity: *I*^2^ = 4%).

#### Anteroposterior

Of the 418 participants included in this study, 198 were assigned to the Pilates group and 210 to the control group.

**Figure 4:**
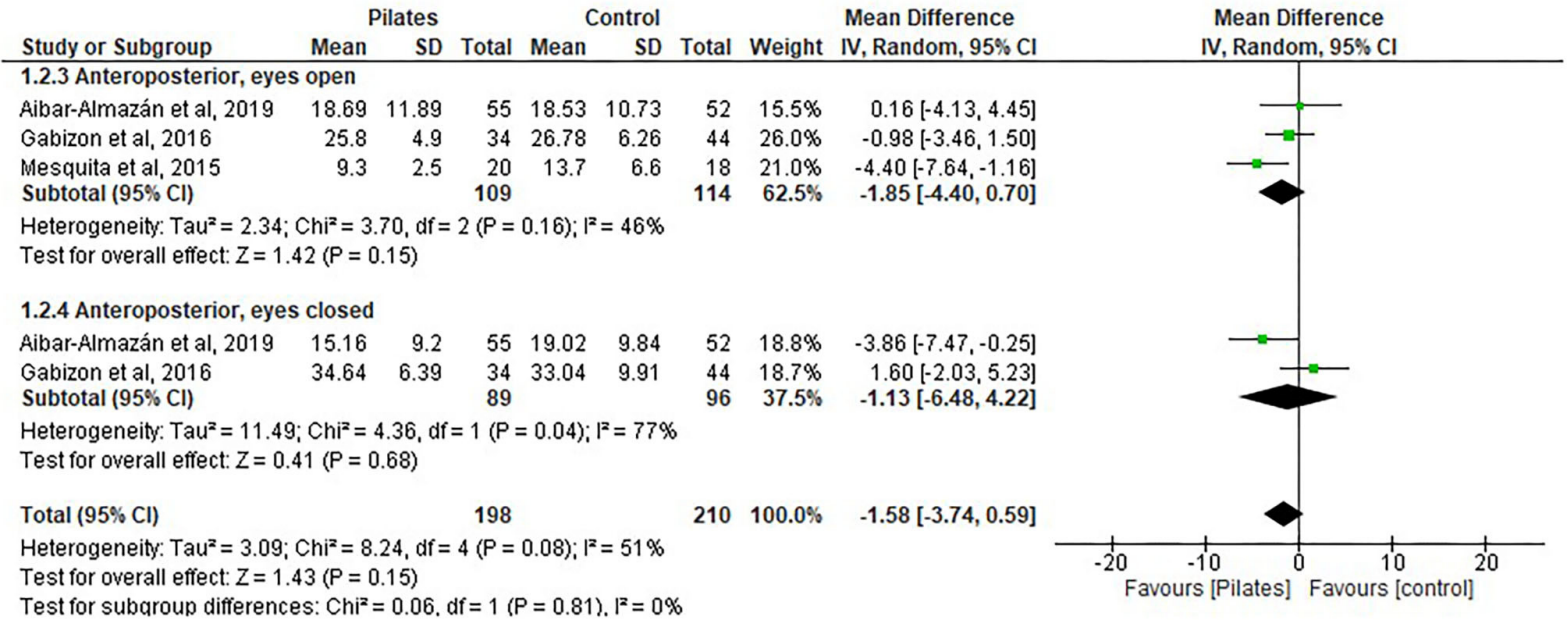
Postural stability-anteroposterior.

The pooled overall balance had a decrease in scores after Pilates and no statistical significance between groups was found: (MD = −1.58, 95% CI, −3.74 to −0.59, *p* = 0.15, heterogeneity: *I*^2^ = 51%) —the postural stability of the subjects increased (see Figure 4).

The following four subgroups were shown separately on the forest plots:

APEO: Three studies (10, 34, 38) included the Pilates group (*n* = 109) vs. the control group with no intervention (*n* = 114). No difference was found between the groups: (MD = −1.85, 95% CI, −4.40 to 0.70, *p* = 0.15, heterogeneity: *I*^2^ = 46%).
APEC: Two studies (10, 34) included the Pilates group (*n* = 89) vs. the control group with no intervention (*n* = 96). No difference was found between the groups: (MD = −1.13, 95% CI, −6.48 to 4.22, *p* = 0.68, heterogeneity: *I*^2^ = 77%).

#### Gait

Two studies (36, 37) included exploratory results. Of the 95 participants included in this study, 48 were assigned to the Pilates group and 47 to the control group. Pilates improved the endurance of the participants, as measured by the increase in the distance of (~30 m, *p* < 0.01) the individual could walk in 6 min (6 MWT) (36) and the increase in velocity of (0.13 m/s) after Pilates measured by the 10-min walking test (10 MWT) (37).

#### DGI

**Figure 5:**
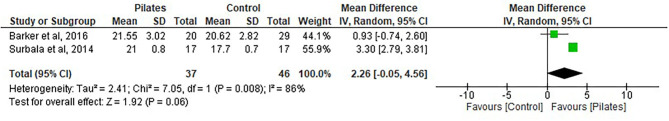
Dynamic Gait Index (DGI).

Two studies were included in this meta-analysis (32, 33), with a total of 37 participants in the Pilates group and 46 participants in the control group (see Figure 5).

Participants showed an increase in their balance and gait scores with Pilates intervention; however, no significant difference was found between groups: (MD = 2.26, 95% CI, −0.05 to 4.56, *p* = 0.06, heterogeneity: *I*^2^ = 86%).

#### TUG Analysis for Pilates vs. Control Groups

**Figure 6:**
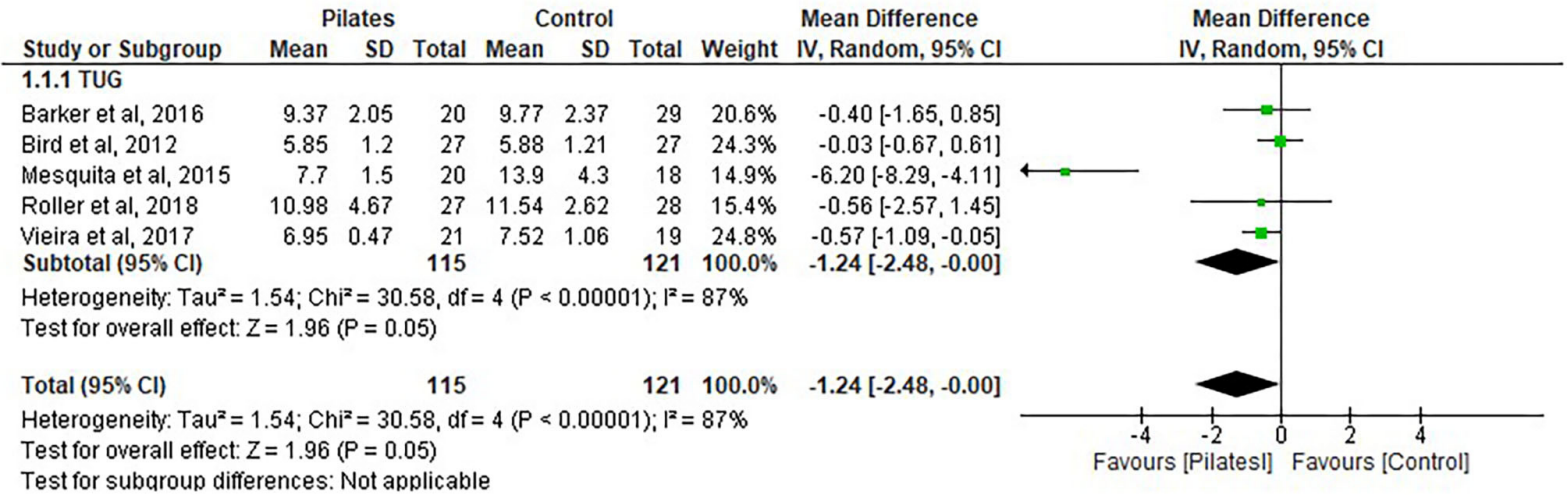
Time Up and Go (TUG1).

Five studies (31, 33, 36–38) were included in the analysis, with a total of 115 participants in the Pilates group and 121 participants in the control group (see Figure 6).

The results show no significant difference between the groups. There was a decrease in the time score (seconds) after Pilates intervention in favor of Pilates: (MD = −1.24, 95% CI, −2.48 to −0.00, *p* = 0.05, heterogeneity: *I*^2^ = 87%).

#### TUG Analysis for Pilates vs. Other Exercise Groups

**Figure 7:**
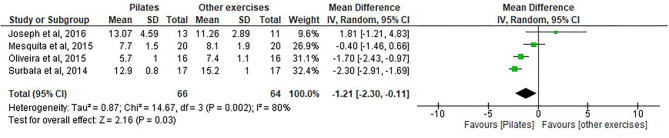
Time Up and Go (TUG2).

Four studies (8, 32, 35, 38) were included in the analysis with a total of 66 participants in the Pilates group and 64 participants in the control group (see Figure 7).

The results show a statistically significant difference between the groups and improvements in functional mobility in favor of the Pilates group by a decrease in the time score (seconds) after Pilates intervention: (MD= −1.21, 95% CI, −2.30 to −0.11, *p* = 0.03, heterogeneity: *I*^2^ = 80%).

#### FRT Analyzed for Pilates vs. Control Group

**Figure 8:**
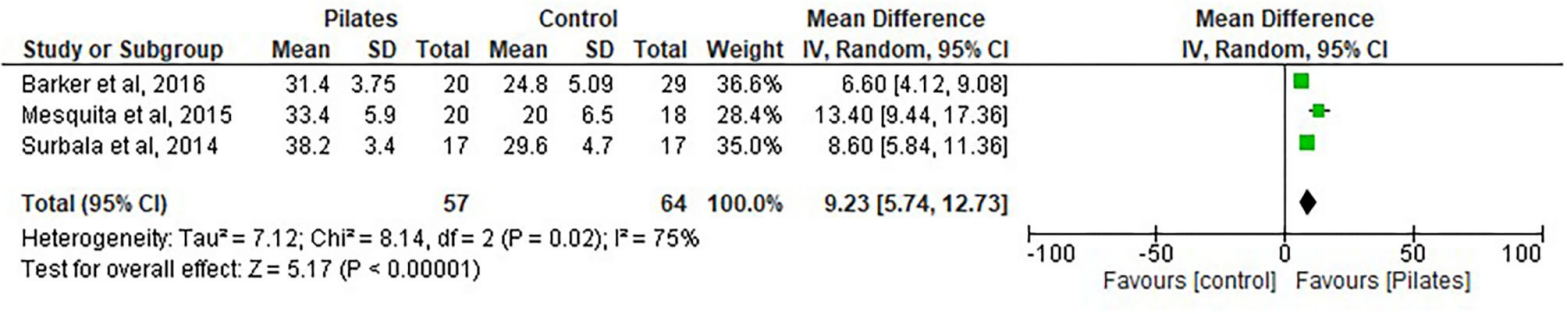
Functional Reach Test (FRT1).

Three studies (32, 33, 38) were included in the analysis, with a total of 57 participants in the Pilates group and 64 participants in the control group (see Figure 8).

There was a statistically significant difference between the groups and an increase in scores after Pilates intervention: (MD = 9.23, 95% CI, 5.74 to 12.73, *p* < 0.00001, heterogeneity: *I*^2^ = 75%).

#### FRT Analyzed for Pilates vs. Other Exercise

**Figure 9:**
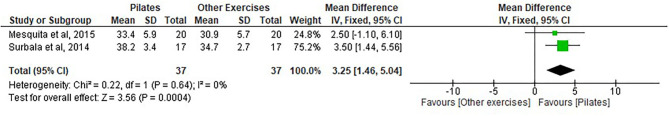
Functional Reach Test (FRT2).

Two studies (32, 38) were included in the analysis, with a total of 37 participants in the Pilates group and 37 participants in the control group (see Figure 9).

There was a statistically significant difference between the groups and an improvement in the Pilates group, as evident from an increase in the score after Pilates intervention (fixed-effects estimation): (MD = 3.25, 95% CI, 1.46 to 5.04, *p* < 0.0004, heterogeneity: *I*^2^ = 0%). There was no evidence of heterogeneity.

## Discussion

The aim of this systematic review and meta-analysis was to explore the effects of Pilates interventions on the following risk factors of falls; mobility, functional mobility, fear of falling, postural stability gait, and falls recorded during the Pilates intervention. To determine if Pilates interventions reduced the risk of falls in healthy older adults, who have maintained functional ability, including participants of both genders, those with a falls history, non-fallers, and individuals who were considered to be sedentary or active. All included studies were RCTs comparing Pilates intervention to control groups. However, only the TUG and FRT measures could be included in the model to compare Pilates to other exercises in the meta-analysis. The main findings of the review show that functional mobility, mobility, gait, postural balance and fear of falling have improved in older participants after practicing Pilates.

### Functional Mobility and Mobility

Participants who undertook Pilates showed greater improvement in mobility/balance than functional mobility in healthy participants when comparing the Pilates group to the control group and to other form of exercises. With regard to FRT, no heterogeneity was found between the two studies presented (32, 38). There were similarities between the number of weeks of intervention (4–6) and in the use of mat Pilates with a ball. Pilates intervention showed sufficient effects to improve mobility/balance in older adults. Barker et al. (19) included exercises involving the standing position with a narrow base, as the standing balance stimulated the vestibular, visual and proprioceptive challenge. The Pilates intervention included a reformer, a trapeze, a Wunda chair, a ball, an elastic band and a foam roller for a 12-week programme with a practice guideline for falls prevention. In Surbala et al. (32), the intervention included mat Pilates with a ball and compared to conventional balance training in fallers, was for a 6-week period, with a frequency of twice per week and a duration of 45 min. In a study by Oliveira et al. (8), Pilates improved functional mobility after two weekly sessions for 12 weeks and included the use of equipment. In the control group, the participants performed static stretching. The exercises in the mat Pilates intervention, which used accessories, were effective for that population. Mesquita et al. (38) found a significant effect in both groups in terms of Pilates and proprioceptive and neuromuscular adaptations in the mat Pilates with accessories class after 4 weeks of intervention in sedentary women. Josephs et al. (35) indicated better improvement in the traditional group (mat with accessories) than in the Pilates group that used equipment. In addition, participants performed home-based exercises after the 12-week programme.

With regard to TUG participants' functional mobility did not improve when compared to the control group. This may be due to the heterogeneity of the methods in the studies, which included both healthy participants and participants with a previous history of falls. Vieira et al. (36) included active participants who might not have had improvement in functional mobility, while Roller et al. (37) included participants with the risk of falls and had not shown much improvement as well.

### Balance Test

In this meta-analysis, Pilates had a positive effect on postural balance in healthy individuals. Considering the mediolateral variable under eyes-open and eyes-closed conditions, there were statistically significant differences between the Pilates and control groups, indicating that Pilates has positive effects and reduces the risk of falls. In an RCT crossover study, Bird et al. (31) also reported a significant improvement in the mediolateral direction under eyes-closed conditions on foam, rather than on a firm surface; however, there was no statistical difference between the groups. It must be emphasized, however, that the washout period of 6 weeks was insufficient to remove the effects of Pilates and after this period, the participants showed neuromuscular adaptation. In contrast, Donath et al. (30) found no significant results among their participants after Pilates training. However, their suggestions for future studies include adaptations in neuromuscular capability, cognitive function and psychosocial health and the use of a three-armed study design. Barker et al. (19) concluded that the control group may have had ceiling effects and a third arm would avoid the effects of Pilates intervention and benefit falls prevention exercises. Furthermore, Gabizon et al. (34) showed no improvement in postural stability after 12 weeks of training. This may be due to the Pilates protocol used, which did not include balance exercises for healthy participants. However, their exercises comprised three levels: 1- traditional Pilates with accessories, 2- Pilates with a Swiss ball, and 3- Pilates involving sitting on a ball using a Theraband (34).

In the present study, postural balance outcomes were evaluated by only comparing the Pilates group to the control group. The quality of the included studies was medium to high on the PEDro scale. Overall, as well as considering the subgroups, the variables analyzed separately for mediolateral directions demonstrated good improvement and good homogeneity and the anteroposterior directions of balance showed heterogeneity. The APEC results should be interpreted with caution because only two studies were included in the meta-analysis for this variable. Further, a random effect estimating for overall for the anteroposterior subgroup was applied due to no homogeneous studies included; however, the forest plot showed a similar weight between them (10, 34). Aibar-Almazan et al.' study had shown great improvement in the decrease score for balance and it was the only study included in this review that was graded at the highest quality. The intervention was a 12-week period of Pilates undertaken twice a week and the authors found improvement in balance confidence, the fear of falling and postural stability; however, the author stated that the best improvement was achieved with variables such as velocity of the COP with eyes open and APEC (10).

Mesquita et al. (38) was deemed to be a medium-quality study. It did not include the intention to treat analysis and allocation was not concealed. Moreover, unblinded assessors and therapists were employed in the study. The intervention period was 4 weeks. The authors found improvement in postural static and functional test performance in both groups of Pilates and neuromuscular facilitation. However, there was no significant difference between the groups (38). It needs to be considered that the consequences of aging affect muscle strength, proprioception, vision and balance of the standing body. Thus, there is a high dependence on the vestibular afferents due to small changes in the body when getting older (41).

In the present study, the balance parameters in the anteroposterior direction under both conditions with eyes opened and eyes closed showed no significant difference between the groups. In another recent meta-analysis, different variables were included to analyse postural balance. Casonatto and Yamacita (20) included six studies in their meta-analysis. Participants performed the balance test on a force platform, with COP directions of ML and AP, area and velocity in the same analysis. Overall, the authors determined SMD to be 0.89 (0.29–1.49) and concluded that the effects of duration and quantity of intervention per week, as well as the quality of the intervention studies, are unrelated to the effects of postural balance.

De Souza et al. (21), another Pilates meta-analysis, also used SMD and included two studies for the total sway area (force platform) and another task—one-leg standing. There was no statistically significant difference between the intervention groups. Low et al. (42) warned that the results of postural control studies can be misinterpreted because the variables are not always analyzed separately or the model employed does not use SMD.

### Gait

In Roller et al. (37), healthy participants improved and increased their speed after a Pilates intervention used a reformer equipment after 4 weeks for once a week to decrease the risk of falls; however, participants who had more functionality had greater improvements in static and dynamic balance. Improved speed was related to increased strength. The study included the 10 MWT test using a timed test to measure participants' speed (43). It was noted that there was an improvement in gait. According to Verghese et al. (28), if each participant walked 10 cm/s, a reduced gait speed is associated with a 7% increase in the risk of falls.

A Pilates study found that participants improved their aerobic capacity and functional exercise by increasing the distance of a 6 MWT after 12 weeks of mat Pilates with accessories such as rubber bands and Swiss balls; however, lower-limb strength could influence the performance test, as the participants were active (36). The study was considered of a lower quality and was unblinded. Moreover, the follow up was inadequate and there was no intention to treat analysis. De Souza et al. (21) evaluated participants after 12 and 24 weeks of intervention and found statistically significant differences between the groups for a 6 MWT (SMD = 2.00, 95% CI 1.44–2.56).

### Fear of Falling and Falls

Participants decreased their concern about falling while doing activities through Pilates intervention compared to control groups. Two studies (10, 11) included healthy older women. Kumar et al. (44). If fear of falling affects an individual's health and social activities, they will experience a decrease in physical abilities and reduce their daily living activities. However, in Badiei et al. (11), the mat Pilates exercises ran for 8 weeks, three times a week. The study included women who were sedentary due to their sociocultural conditions and limited the women to practiced exercises. In Aibar-Almazán et al. (10), the Pilates exercise ran for 12 weeks, twice weekly and used accessories such as resistance bands, rings and balls. The study was considered to be of a high quality, was blinded, had the intention to treat and had a sample size greater than the other studies.

Fear of falling is more apparent among individuals who have experienced previous falls and there is an association with reduced gait speed, stride length, double support time (45, 46).

In the present study, there was a lack of data for fallers and no fallers, these dichotomous variables were not analysis the risk of falls; there were only two studies that included the number of falls that occurred during this period—Barker et al. (33) evaluated the risk factors, which included falls and injuries; however, the results showed that there was no significant difference between the Pilates and control groups for the rate of falls. Barker et al. (33) and Josephs et al. (35) showed that there was a reduction in scores, favoring the Pilates group. Four studies (10, 11, 34, 37) did not include data on the number of falls occurring during their intervention programme. Roller et al. (37) reported that the number of falls during the intervention programme was missing. A further meta-analysis found that Pilates prevented falls (19). However, that study included only one study in the analysis; hence, the results could have been misinterpreted.

### Studies' Recommendations for Fall Prevention

Barker et al. (33) recommended specific training with a physical therapist, including exercises in a standing position, to reduce the risk of falls. Bird et al. (31) noted an improvement in the mediolateral directions of balance and participants' dynamic balance showed the intervention's positive implications for physical fall risk factors in older adults. Roller et al. (37) noted that more studies are needed to assess the effects of Pilates using Reformer equipment as related to balance, gait and falls risk in older adults. Further specific exercises focused on balance in upright postures for standing, moving and carriage were advised.

### Limitations of the Review

This systematic review and meta-analysis have some limitations. The study included only RCTs, full-text versions and articles published in English. However, the study included studies that ranged in quality from medium to high. It was not possible in this study to analyse Pilates interventions in comparison to other exercise groups in terms of the most selected measures, and not all studies included focused on falls. Some low-quality studies included other forms of exercise, and the studies included focused on the outcome measures to decrease fall risk, where the primary outcomes of this study were functional mobility and postural balance.

There is still insufficient evidence in the literature to state conclusively that Pilates is an effective form of exercise to prevent falls. Concerning the number of falls and the number of fallers reported during the intervention programme, there were other limitations related to the low number of studies included. Moreover, meta-analyses are also dependent on heterogeneity among studies, such as in the clinical implications, Pilates methods and test measures used. However, according to Casonatto and Yamacita (20), the heterogeneity of Pilates intervention methods (frequency, duration, and quality of studies) previously mentioned was unrelated to the effects on postural balance. This study followed the PICO criteria to focus on measures to decrease fall risk. In addition, due to the lower number of RCTs in Pilates, it was preferred to include all types of Pilates interventions.

### Implications of the Results for Practice

There was no improvement in dynamic gait index and it was not possible to analyse the spatiotemporal parameters of gait due to the lack of research data in this area. Thus, the spatiotemporal parameters of gait were lacking in the RCT Pilates studies. Most of RCT Pilates studies that have analyzed gait parameters have considered neurological participants for the inclusion criteria. Further, Pilates studies that have included gait parameters did not have groups comparison for healthy participants. With these gaps this study did not include the spatiotemporal parameters of gait outcomes. However, we have included the clinical assessments (MWT) for gait. Moreover, it was not possible to analyse gait due to the lower number of studies included.

### Future Research

This systematic review and meta-analysis included only RCTs, meaning the quality and rigor of the methodology were increased. Further outcomes and more evidence from RCTs must be provided. Future studies should consider the number of falls and of faller participants among their primary outcomes during the intervention programme. In addition, studies should include a diary of falls for everyone or an electronic app to monitor the daily falls of each participant. Future studies should investigate any benefits in saving cost of groups and classes with a supplementary home-based Pilates intervention or an individual home-based exercise. May the risk of bias increasing; however, it is difficult to blind the participants and the instructor. Studies with a longer follow-up period are warranted.

## Conclusion

There is some evidence to suggest that Pilates reduces certain risk factors for falls in healthy older adults. Pilates intervention, when compared to control groups, was shown to improve functional mobility, general mobility, postural balance, gait, and fear of falling of healthy older adults, which may decrease their risk of falls. Pilates intervention, when compared to control groups, showed no improvement in functional mobility than other exercises. Pilates did, however, show greater improvement in mobility than other exercises. It is evident that 4–6 weeks of Pilates intervention without equipment had positive results on general mobility. Pilates was found to improve fear of falling and postural stability in the mid-lateral directions with eyes open and closed, thereby potentially decreasing the risk of falls. Other evidence has shown conflicting results with regard to balance and postural stability including different measures. Further robust studies are needed to evaluate the number of falls and to incorporate falling participants into a Pilates intervention programme with longer follow-up. The intervention programme, including different methods, has implications for future research with regard to the use of mat Pilates, equipment and the number of weeks required for the Pilates intervention.

## Data Availability Statement

The original contributions presented in the study are included in the article/supplementary files, further inquiries can be directed to the corresponding author/s.

## Author Contributions

LD: study design, database management and search strategies, screened studies for inclusion, extracted data, quality of data extraction, and data extraction for meta-analysis, analysis, acquisition, or interpretation of data, and drafting of the manuscript, wrote and reviewed the manuscript, critical revision of the manuscript for important intellectual content. CM: designed the review, screened studies for inclusion, extracted data, checked quality of data extraction, contributed to writing and editing the review, advised on the review, and approved final review prior to publication, critical revision of the manuscript for important intellectual content. AS: study design and reviewed the manuscript. All authors contributed to the article and approved the submitted.

## Conflict of Interest

The authors declare that the research was conducted in the absence of any commercial or financial relationships that could be construed as a potential conflict of interest.

## Publisher's Note

All claims expressed in this article are solely those of the authors and do not necessarily represent those of their affiliated organizations, or those of the publisher, the editors and the reviewers. Any product that may be evaluated in this article, or claim that may be made by its manufacturer, is not guaranteed or endorsed by the publisher.
